# The Breeding Information Management System (BIMS): an online resource for crop breeding

**DOI:** 10.1093/database/baab054

**Published:** 2021-08-20

**Authors:** Sook Jung, Taein Lee, Ksenija Gasic, B. Todd Campbell, Jing Yu, Jodi Humann, Sushan Ru, Daniel Edge-Garza, Heidi Hough, Dorrie Main

**Affiliations:** Department of Horticulture, Washington State University, 45 Johnson Hall, Pullman, WA 99164, USA; Department of Horticulture, Washington State University, 45 Johnson Hall, Pullman, WA 99164, USA; Plant and Environmental Sciences Department, 171 Poole Agricultural Center, Clemson University, Clemson, SC 29634, USA; Coastal Plains Soil, Water, and Plant Research Center, USDA-ARS, 2611 West Lucas St., Florence, SC 29501-1242, USA; Department of Horticulture, Washington State University, 45 Johnson Hall, Pullman, WA 99164, USA; Department of Horticulture, Washington State University, 45 Johnson Hall, Pullman, WA 99164, USA; Department of Horticulture, University of Auburn, 287 CASIC Building/120 Funchess, Auburn, AL 36849, USA; Centre for Horticultural Science, The University of Queensland, Brisbane St Lucia, Brisbane, QLD 4072, Australia; Department of Horticulture, Washington State University, 45 Johnson Hall, Pullman, WA 99164, USA; Department of Horticulture, Washington State University, 45 Johnson Hall, Pullman, WA 99164, USA

## Abstract

In this era of big data, breeding programs are producing ever larger amounts of data. This necessitates access to efficient management systems to keep track of cross, performance, pedigree, geographical and image-based data, as well as genotyping data. In this article, we report the progress on the Breeding Information Management System (BIMS), a free, secure and online breeding management system that allows breeders to store, manage, archive and analyze their private breeding data. BIMS is the first publicly available database system that enables individual breeders to integrate their private phenotypic and genotypic data with public data and, at the same time, have complete control of their own breeding data along with access to tools such as data import/export, data analysis and data archiving. The integration of breeding data with publicly available genomic and genetic data enhances genetic understanding of important traits and maximizes the marker-assisted breeding utility for breeders and allied scientists. BIMS incorporates the use of the Android App Field Book, open-source phenotype data collection software for phones and tablets that allows breeders to replace hard copy field books, thus alleviating the possibility of transcription errors while providing faster access to the collected data. BIMS comes with training materials and support for individual or small group training and is currently implemented in the Genome Database for Rosaceae, CottonGEN, the Citrus Genome Database, the Pulse Crop Database, and the Genome Database for Vaccinium.

Database URLs: (https://www.rosaceae.org/), (https://www.cottongen.org/), (https://www.citrusgenomedb.org/), (https://www.pulsedb.org/) and (https://www.vaccinium.org/)

## Introduction

Breeders produce large amounts of data, including phenotype, genotype, cross, germplasm and pedigree data. Database systems are needed to upload, store and easily query the data to make accurate and timely breeding decisions. For example, the breeders need to (i) select elite offspring based on their phenotypic performances, (ii) select superior parents based on the performance of their offspring, (iii) evaluate the effects of environment on crop performance based on data collected in multiple years and locations, (iv) identify correlations between traits and (v) conduct DNA-informed breeding based on marker data on key loci or across the genome. Breeders typically use book-based systems, Excel spreadsheets, R data files or Microsoft Access to keep track of their data, all of which can require breeders to spend significant effort and time to find the data that they need. Proprietary software, such as AgroBase (https://www.agronomix.com/AGROBASE.aspx), for managing breeding data is available but it is costly. In addition to the initial cost, proprietary software often requires an extra fee to add more data types such as a new trait descriptor. As the recent survey (1) shows, many public breeding programs in the USA reported budget shortfalls that make it difficult for them to use proprietary software for data management. U.S. Breeding programs reported, on average, devoting 2.78 full-time equivalent employees to plant breeding research in the most recent fiscal year and almost 80% of programs reported annual budgets of US $400 000 or less (1). The breeding management system (BMS) from the International Maize and Wheat Improvement Center is available as a single-user free download or as a multi-user program with pro features and support services for a fee (https://www.integratedbreeding.net/). A server version is also available with a fee for users with data-sharing requirements. The code behind Sol Genomics Network (2) is made available as Breedbase (https://breedbase.org/). Breeding databases have been built by Breedbase for crops such as cassava (https://cassavabase.org), sweet potato (https://sweetpotatobase.org), banana (https://musabase.org) and rice (https://ricebase.org).

In order to provide breeders with a free management system that allows individual breeders to have complete control of their own private breeding data with an option to easily integrate publicly available data in the community crop database, we have developed the Breeding Information Management System (BIMS) within community databases. The functionality of BIMS to import publicly available data is powerful since breeders can easily import phenotype data of accessions within the community database and integrate with their data to compare the performance and use as potential parents. In addition, since breeders more commonly use DNA information in their breeding program, data such as genomic and genetic positions of SNPs and genotype data of elite cultivars can be extremely useful when integrated with their private data. For example, they can search their private genotype data by genome locations of SNPs without loading that data into their BIMS if the SNP location data are available in their community database.

BIMS was developed as an extension module of Tripal (3), an ontology-based, open-source toolkit for the construction of online biological databases. Tripal has been widely used in building databases with at least 31 public genomic and genetic databases using this platform (3). Since Tripal is open-source and modular software, any site developer can create their own extension modules to share with other Tripal users. Over 40 extension modules are available on the Tripal website (http://tripal.info/) that can be used by database developers. Some examples include the Tripal MegaSearch module, which allows interactive and customizable query and download (4); the TripalMap module, which displays genetic map data (5) and the Tripal Analysis Expression module for loading, annotating and visualizing NCBI Biomaterials and expression data (6). As technology advances, new data types and analysis methods emerge all the time. BIMS, because it resides within a community database in a platform where developers worldwide contribute new search, visualization and analysis modules, has significant potential for breeders to integrate new types of data into their database and to use new analysis tools with their breeding data.

BIMS is currently available in the Genome Database for Rosaceae (7), CottonGEN (8), Citrus Genome Database (9), Pulse Crop Database (10) and the Genome Database for Vaccinium (11). The breeders of the 26 crops that are covered by these databases can create their private breeding program using BIMS. BIMS can also be used by a group of breeders within a collaborative project to share data. In addition, BIMS is used as a tool for the scientists to explore public breeding data in the community databases referenced above. With the source code made available, BIMS can be installed in any database built in Tripal, such as TreeGenes (12), KnowPulse (13), Curcurbit Genomics Database (14), PeanutBase (15) and CarrotDB, thereby providing future opportunities for adoption by more breeders. Any other crop community can also build their genomic, genetic and breeding database using the Tripal core module and BIMS. The BIMS module and user documentation can be downloaded from GitLab: https://gitlab.com/mainlabwsu/bims.

This paper describes how breeders can use BIMS to manage their private breeding data. It details how to access BIMS to create accounts, import data, search and analyze data, edit data and customize BIMS to fit their breeding program. It also points to future development plans for this breeding resource.

### Overview

BIMS is an online tool that allows users to explore the publicly available breeding data in the host community databases while also providing a data management solution for private breeding programs. The BIMS website (https://www.breedwithbims.org) has information about BIMS including training materials, news, work completed, work in progress, webinars, presentations, mailing lists, BIMS manual, links to various community databases and links for developers to access the module code.

BIMS provides individual breeders with a secure and comprehensive online BMS that allows them to store, manage and analyze their private data, which can then be fully integrated with publicly available genomic, genetic and breeding data within Tripal databases. The BIMS tool supports the use of Field Book (16), an Android app for collecting phenotype data in the field. Field Book is available through the Google Play Store. In the following sections, we provide information on how to use the current version of BIMS.

**Figure 1.**
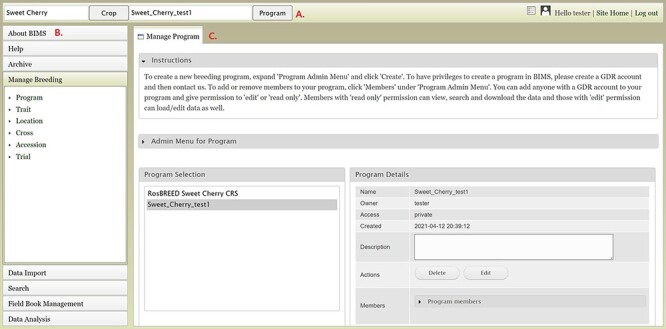
Main areas of the BIMS interface. A. The header region that displays the current crop and program on the left and links to configuration page, account page and home page on the right along with the user name. B. The accordion menu on the left that allows users to switch between various sections of the BIMS C. The tab region where users primarily interact with BIMS.

There are three main areas in the BIMS interface. The first part is the header region (Figure 1A), which displays the selected crop and program. Here, users can change the crop or program, and there are links to the configuration page, account page and the homepage of the community database (Site Home). The header also displays the username if the user is logged in. If the community database has only one crop, the header region will not show the crop section. The second part of the interface is the accordion menu on the left side (Figure 1B). This menu allows users to switch between various sections of BIMS. The last region is the tab region (Figure 1C), and this is where users primarily interact with BIMS. Each tab in BIMS has an Instructions section that can be expanded by clicking on it. The Instructions section provides information on how to use that tab. Without logging in, users can choose a crop and a public BIMS program to view data and explore BIMS. The About BIMS and Help menus provide various information sources including links to the BIMS manual and mailing list.

### Creating a new breeding program

**Figure 2.**
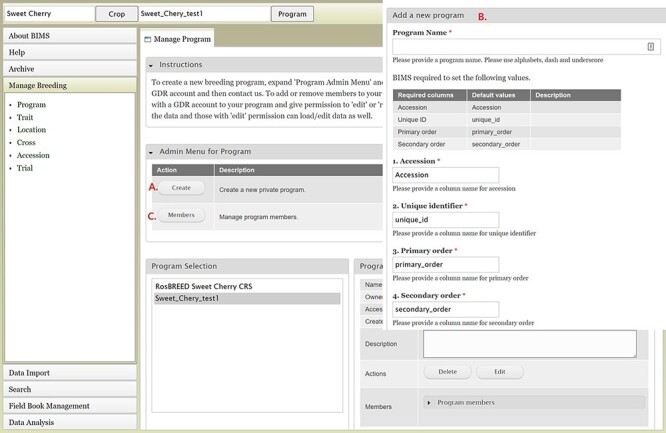
BIMS interfaces for creating, editing and managing programs. A. Manage Program tab showing the list of programs, the details of the highlighted program and admin menu for managing program. B. Add Program tab where users can enter the new program name and modify the four column names required in the BIMS template and Field Book to match their dataset. C. Members tab where users can add other account holders as members of the breeding program with edit or read-only privilege.

To create a breeding program, users need to first create an account in the community database and then request breeder privileges through the contact form. Users with breeder privileges can create a new breeding program in BIMS. If the community database has multiple crops, the first step in creating a new program is to select the appropriate crop. When the new crop is selected, the Manage Program tab will open and then users can open the Admin Menu for Program section. Users can click Create to make a new program (Figure 2A). In the Add Program tab (Figure 2B), users can enter the new program name and modify the four column names required in the BIMS template and Field Book to match their dataset. Accession refers to the name of a breeding line with a unique genotype, such as ‘Fuji’ or ‘Cosmic Crisp’ in the case of an apple crop. The three other columns—unique identifier, primary order and secondary order—are required columns in Field Book. A unique identifier is used internally to associate data with a specific entry and should be unique within the dataset. Primary order and secondary order are used to represent the positions of the plants in the field or orchard. The column names can be changed, and they are often used for row, column, range and plot. After creating the program, users will have the option to add other account holders, such as members of their breeding program, with edit or read-only privilege (Figure 2C). Once the program is created then data can be loaded to the program.

### Importing data

**Figure 3.**
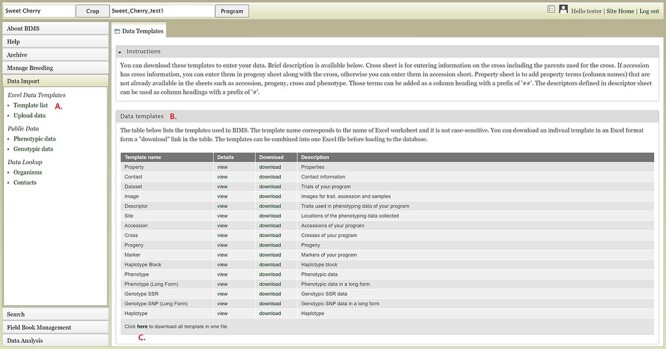
BIMS interface for downloading data templates. A. Template list section under Data Import menu. B. Data templates section that lists all the data templates with hyperlinks to view and download. C. Link where users can also download all the templates as a single Excel file.

**Figure 4.**
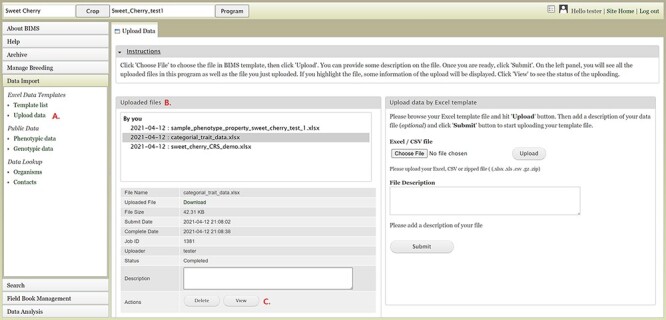
BIMS interface for uploading data in templates. A. Upload data link under Data Import section. B. Uploaded files section In the Upload Data tab that shows the name of the files that have been submitted or uploaded. C. View link that opens a new tab that shows the progress of an upload job.

To import data into BIMS, the data need to be in the BIMS templates that are available for download in the Template list under the Data Import section on the left-hand accordion menu (Figure 3A). Phenotype data can also be imported to BIMS using exported files from Field Book. The Data Templates tab lists all the templates with hyperlinks to view and download the template (Figure 3B). Users can also download all the templates as a single Excel file (Figure 3C). Once the data have been entered into the templates, users can load the data using the Upload data link (Figure 4A) under the Data Import/Excel Data Templates section. In the Upload Data tab, the file name will appear in the Uploaded files section after a file has been submitted (Figure 4B). To view the progress of an upload job, users can click on the file name and the View button (Figure 4C).

**Figure 5.**
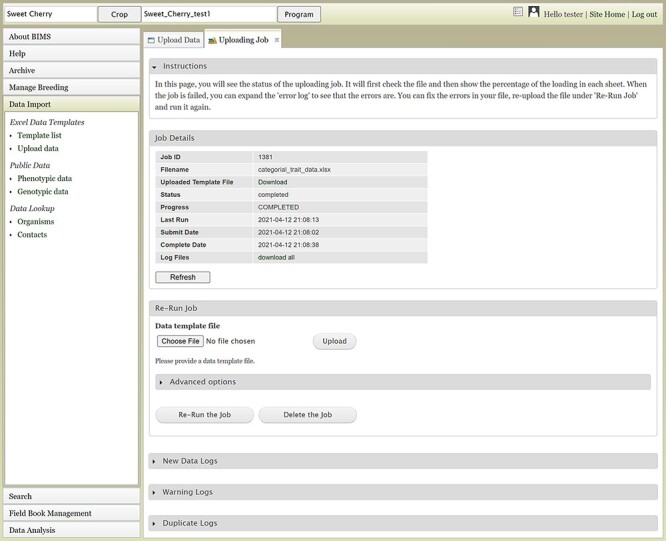
The Uploading Job tab that shows the progress of an uploading job. When the job is completed or failed, the tab displays various logs such as New Data Log, Warning Log, Duplicate Log and Error Log.

The Uploading Job tab will show the Job Details table (Figure 5), which indicates the job progress. BIMS also provides links to log files on the Uploading Job tab. When a job successfully loads, there can be three types of logs: New Data Logs, Warning Logs and Duplicate Logs. New Data Logs describe the new data that was loaded. Warning Logs indicate if any data were skipped due to incorrect column headers or typos, but these data were not critical for BIMS to load data, so the job is still completed. It is recommended for users to look at the Warning Logs to make sure that data were not omitted accidentally. The Duplicate Logs display what data were a duplication of data already present in BIMS. If an upload job fails due to an error, an Error Log section will also be available on the Uploading Job tab. Users can use the information in the Error Log to modify the original file. To re-run a failed job, users can use the Re-Run Job section on the Uploading Job tab. If the error was not due to a data error in the template (for example, a typo in the configuration setting), it is best to go back and start the loading process again instead of using the Re-Run Job section.

**Figure 6.**
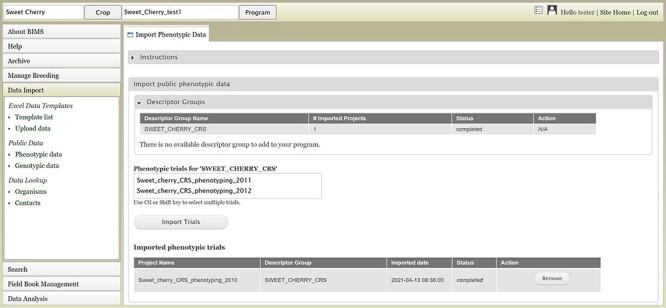
BIMS interface for uploading public phenotype data. Users can import any publicly available dataset to their program.

Users can also upload publicly available phenotype or genotype data available in the community database to their program. Figure 6 shows the tab where users can view and import public phenotype data for their crop. The tab lists trait descriptor groups and then phenotypic data for each descriptor group. The table at the bottom shows the status of imported phenotypic data. After the phenotype data are imported, users can view and search the data in each appropriate section of BIMS

### Entering data into template

**Figure 7.**
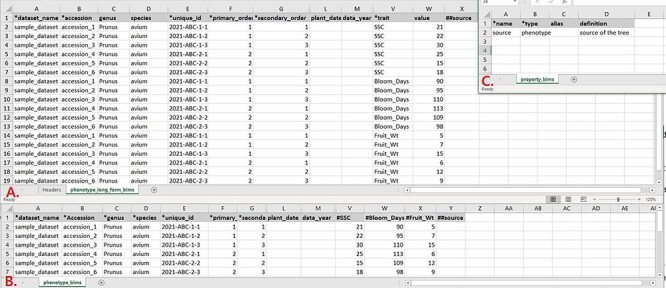
Example data templates in Excel. A. A template for phenotype data where trait names are entered in a column. B. A template for phenotype data where trait names are entered as a column heading. C. A template where users can enter data for user-defined column headings.

Data templates are Excel files that contain metadata type as headings where data users can enter data to be loaded into the BIMS database. In the current version of BIMS, templates are available for each of the following data types: property, contact, dataset, image, descriptor, site, accession, cross, progeny, marker, haplotype block, phenotype, SSR genotype, SNP genotype and haplotype. There can be multiple templates for some data types. Figures 7A and B show two templates that belong to the phenotype template type, an example of a template type that has multiple templates.

In phenotype_long_form_bims (Figure 7A), there is a trait column where the trait descriptor names are entered. Right next to it is a value column where the trait value is entered. Each row contains one accession name, one trait name and the value for the trait and accession combination. In this way, the number of the rows is the product of the number of trait descriptors and the number of accessions. For example, if 10 traits are measured for 50 accessions, a total of 500 rows are entered. In phenotype_bims (Figure 7B), trait descriptor names are entered as column headings with # as a prefix. Each row contains one accession name and all trait values. In this way, the number of the rows is the same as the number of the accessions. For example, if 10 traits are measured for 50 accessions, a total of 50 rows are entered. Having two different templates are convenient for users since the output of some phenotyping software is similar to the wide form and others to the long form.

As shown in Figure 7, the column headings with * prefix in the templates are required fields. Some of the column headings, such as dataset_name, accession, evaluator, trait and site_name, have associated data that need to be entered in a separate sheet: dataset_bims, accession_bims, contact_bims, descriptor_bims and site_bims. The data in these columns of the phenotype sheets should match the data in their respective sheets. For example, if an accession name in a phenotype sheet is ‘Cosmic Crisp’, it should be entered in the accession sheet as ‘Cosmic Crisp’ with associated data. Once the data are loaded into the BIMS database; however, the same data does not need to be in the template. For example, once all the accessions used in the trial are loaded along with the associated trial name, site, trait name and contact information using appropriate sheets, users can use only the phenotype sheets to load new phenotype data.

In the phenotype sheets (Figure 7), there are additional columns to those required so that users can load appropriate data. When there is a need for custom columns not already found in the phenotype sheets, however, users will need to enter column headings with a prefix ##. The headings then need to be entered with associated data in the property_bims sheet (Figure 7C). The custom property columns can be added in cross_bims and accession_bims sheets as well. Once the data are loaded, custom properties can be selected as search categories in BIMS.

### Manage breeding section to view, create and edit data

**Figure 8.**
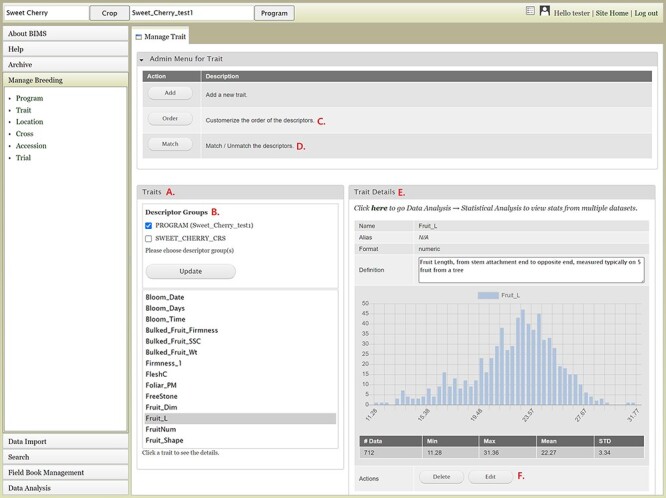
BIMS interface to view, create, or edit data for Trait. A. Trait section in Manage Trait tab where all the traits in the program are listed. B. Descriptor groups section where users can choose one to view the traits that belong to the descriptor. C. Order button under Admin Menu for Traits that opens up a new tab to change the order of trait descriptors in Traits section. D. Match button under Admin Menu for Traits that allows users to merge the same descriptor in different descriptor groups when they represent the same descriptor with the same unit and phenotyping method. E. Trait Details section that shows the details of the trait descriptor along with a graph that shows the distribution of the trait values in the entire data loaded to BIMS along with the statistical values. F. The Edit button in the Trait Details section that opens a new tab where users can edit the details of the trait.

The Manage Breeding section of the accordion menu lets users view, create or edit data for Trait, Location, Cross, Accession and Trial, in addition to Program. When users click Trait under Manage Breeding in the accordion menu, the Manage Trait tab opens on the main page. In the Manage Trait tab, users can see all the trait descriptors in the Traits section (Figure 8A). When there is imported public phenotype data, the descriptor groups of both public data and private data are displayed (Figure 8B). The order of trait descriptors in the Traits section can be modified by clicking the Order button in The Admin Menu for Trait section (Figure 8C). The Match button (Figure 8D) allows users to merge the same descriptor in different descriptor groups when they represent the same descriptor with the same unit and phenotyping method. This allows users to view, search and analyze the phenotype data of the combined dataset. The Admin Menu for the Trait section also has an Add button to add a new trait manually without using a template.

Once users click one trait descriptor in the Traits section, the Trait Details section on the right side will show details of the trait descriptor along with a distribution of the trait values and statistical summary of the full dataset loaded to BIMS (Figure 8E). At the bottom of the Trait Details section, Delete and Edit buttons are available. The Delete button should be used with caution since it will delete all the trait values that are associated with the trait descriptor as well as the details of the trait descriptor. The Edit button will open a new tab called Edit Trait (Figure 8F) to allow users to edit the details of the trait, such as name, data unit, default value and description. Similar functionalities such as adding, viewing, editing and deleting data are available in the Manage Location tab. When a location has latitude and longitude data, a Google Map button will be available under the Location Details section. In the Manage Cross tab, users can manually add or delete a cross or view details of a cross including trait data for a specific cross. In the Manage Accession table and the Manage Trial tab, users can manually add, edit, delete and view details of an accession or a trial.

### Searching and editing data

**Figure 9.**
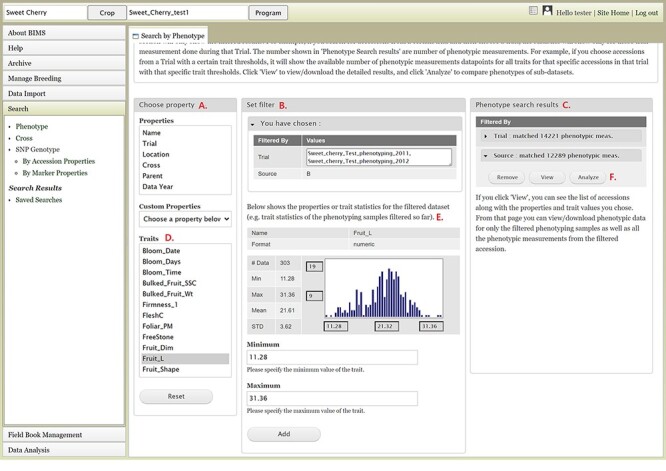
BIMS interface to search phenotype data. A. Choose a property section where users can filter the dataset by choosing various properties or traits. B. Set filter section that is populated with options for the data type when users choose a property or a trait. C. Phenotype search results section that shows the selected filter. D. Trait list in the Choose property section. E. Diagram of the trait value distribution for the previously filtered dataset. F. Remove, View, and Analyze buttons in the Phenotype search results section.

**Figure 10.**
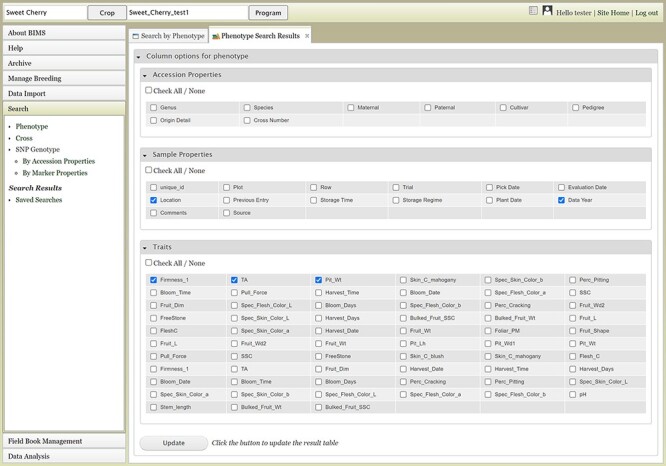
Upper section of the Phenotype search results tab where users can add more columns in the result table.

**Figure 11.**
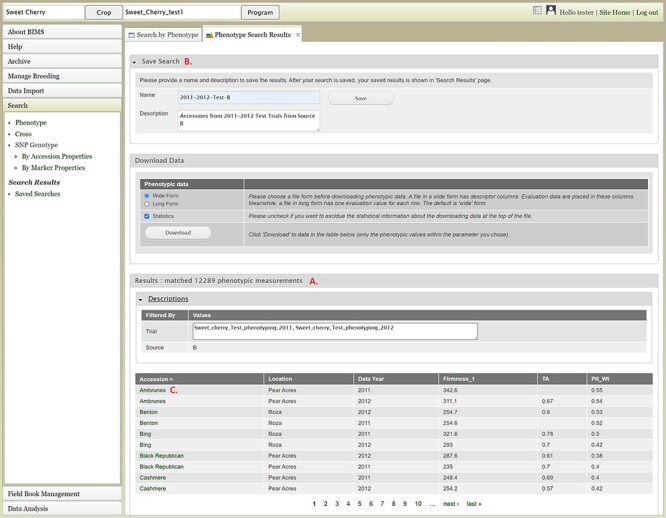
Lower section of the Phenotype search results tab. A. Result table section. B. Save Search and Download Data section. C. Any data under Accession column of the result table has hyperlinks to open a new tab to view the phenotype details of the specific sample.

**Figure 12.**
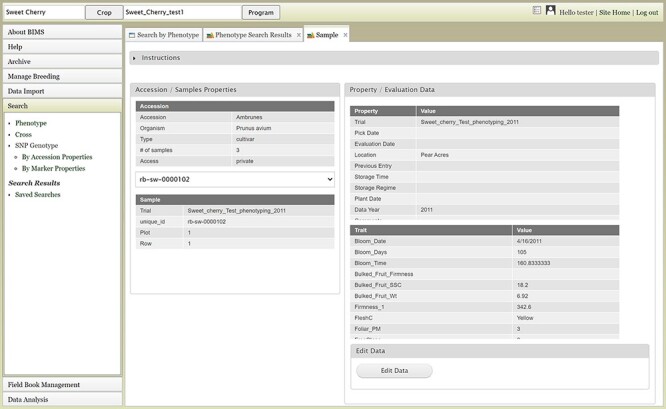
Sample tab where users can view and edit the phenotype details of a specific sample.

The Search section of the accordion menu allows users to search phenotype, genotype and cross data. When users click Phenotype under Search, the Search by Phenotype tab appears. This tab has three sections: Choose property, Set filter and Phenotype search results (Figure 9). The first step to start a search is to select a property or trait under the Choose property section (Figure 9A). For example, users can choose Trial under Properties. After the property is selected, the Set filter section will populate with options for that data type (Figure 9B). After selecting the options, users can click the Add button. The selected filter will then appear in the Phenotype search results section (Figure 9C). Users can further filter the search results by adding another property or trait under the Choose property section. For example, users can restrict data using one of the Custom Properties that they introduced in the template. Users can further refine the search by adding thresholds for a trait (Figure 9D). The Set filter section will show the data that are relevant to the previously chosen filter. For example, the statistics shown for the Fruit_L trait at this step (Figure 9E) is for the accessions from the trials chosen in the previous search. Users can set the minimum and maximum values of this trait and then click Add, which then adds the new filter to the Phenotype search results section. Any selected filters can be removed with the Remove button under each filter (Figure 9F). To view the search results and to generate a file to export, users can click on the View button (Figure 9F). To analyze the filtered dataset, users can click on the Analyze button (Figure 9F). When the View button is clicked (Figure 9F), a new tab called Phenotype Search Results opens (Figure 10). This tab includes a section called Column options for the phenotype (Figure 10) and a Results section that features a table of the search results (Figure 11). Under the Results section, the Descriptions menu can be expanded to show the filters that were used to generate the initial table. Users can add more information to the table before exporting the data under the Column options for the phenotype section (Figure 10). There are three menus—Accession Properties, Sample Properties and Traits (Figure 10)—where additional fields can be selected to add these data to the result table. After selecting the desired fields, followed by selecting Update, users can view the reflected changes that match their selections in the table under the Results section (Figure 11A). Users can also save the search in the Save Search section or download the data in the Download Data section (Figure 11B). Saved search results can be viewed and downloaded in the left-hand accordion menu: Search > Search Results > Saved Searches. Saved search results can be further used as accession lists for statistical analyses (Data Analysis > Phenotypic Data > Statistical Analysis in the accordion menu) and to generate new input files for the Field Book app (Field Book Management > Generate input file). These downstream features are explained further below. Users can click the Accession name (Figure 11C) from the table under the Search Results tab, which then displays the phenotype details of the selected sample in a new (separate) Sample tab (Figure 12). By clicking Edit Data in the Sample tab, users can edit various properties and trait values (Figure 12). These functionalities are especially useful if quick decisions need to be made—such as, for example, during the growing/ripening season in the tree fruit breeding program to choose the best accession within a specific ripening date to propagate on rootstock via grafting/budding. Prior to BIMS Excel and/or Microsoft Access, filtering options would be used to find the information needed. That requires opening several Excel files, sometimes up to 6 or 7 depending on for how many years field data were available, and filtering or using query in Microsoft Access to pull desired information. With BIMS search functionality, all data, even from the current season, could be summarized within a fraction of the time and confident decisions can be made.

**Figure 13.**
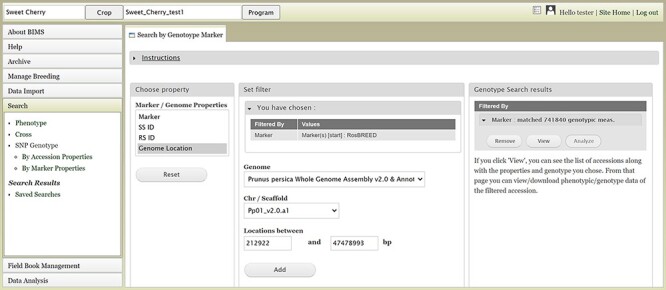
BIMS interface to search genotype data. Users can search SNP genotype data by accession properties or marker properties.

**Figure 14.**
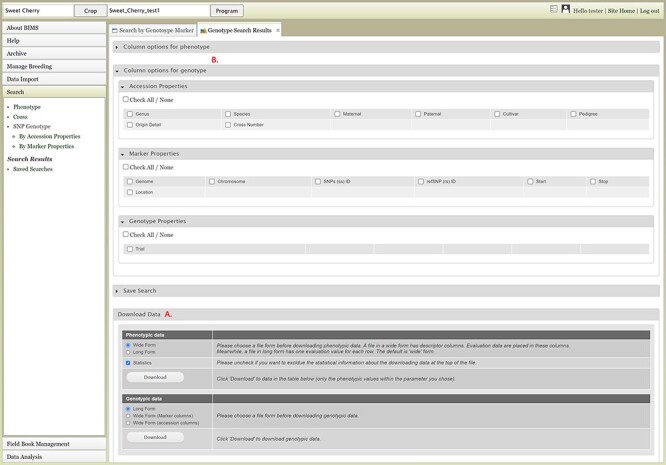
Genotype Search Results tab. A. Download Data section where users can download phenotype data as well as genotype data when the data is available. B. Column options section where users can add columns either for phenotype or genotype data. C. Link to the account page where users can access the downloaded data files.

Users can search SNP genotype data by accession properties or marker properties. Marker properties used as default search categories include marker name, SS ID, RS ID, and genome location of the SNPs (Figure 13). In the Genotype Search Results tab, users can download the phenotype data as well as genotype data of the accessions filtered by the genotype search (Figure 14A). Users can add columns to the downloadable file of both phenotype and genotype data (Figure 14B). Downloaded data files can be accessed in the account page that can be accessed in the top section of each BIMS page (Figure 14C).

### Data analysis

**Figure 15.**
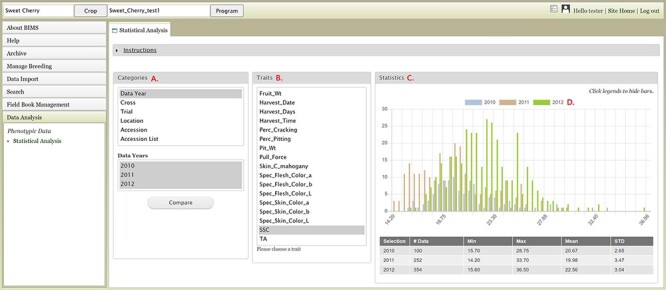
BIMS interface where users can compare trait values among different datasets. A. Categories section where users can choose multiple datasets B. Traits section to choose a trait statistics of the chosen dataset. C. Statistics section where users can view a graph with the distribution of the trait values for each dataset. D. Legends can be deselected to view subsets.

The Data Analysis section of the accordion menu lets users further analyze phenotype data. This section allows users to choose multiple datasets, using categories, and compare the trait values among the multiple datasets. When users click Statistical Analysis under Data Analysis, the Statistical Analysis tab appears (Figure 15). The tab has three sections: Categories, Traits and Statistics. Users can select a category under the Categories section and then choose multiple datasets in the second box that pops up once a category is selected (Figure 15A). Categories include the Accession List, which is the list of accessions that users saved in the Search section. After choosing multiple datasets, users can click the Compare button. A bar will appear to show the computation progress. When the computation is done, users can select a trait in the middle section (Figure 15B). The Statistics section on the right will show a graph with the distribution of the trait values for each dataset (Figure 15C). A table below the graph will show the statistical values for each dataset. Users can click the legend to deselect some of the dataset fields to view subsets (Figure 15D). Users can also access the Statistical Analysis from the Search page and the analysis will be done on the dataset that users created on the Search page (Figure 9F).

### Field book management

The Field Book Management section of the accordion menu provides functionality to generate input files for Field Book or load data to BIMS using the Field Book output files. Two types of files, the field file and trait file, can be generated using data in BIMS to load to the Field Book App.

The *Configure Field Book* section allows users to pre-select columns that can be included in the field file. By default, the three columns required in the field file—a unique identifier, primary order, and secondary order, as well as accession—are downloaded in the field file. The Field Book Configuration page allows other columns to be included in the Field Book input file so that Field Book users can see those on the main screen of the Field Book. This additional information displayed on the screen, such as parents or a certain trait value, helps breeders to distinguish the accession they are phenotyping. The additional information includes traits, accession properties and sampleproperties.

The Trait link under the *Generate input file* section opens a page where it lists all the traits stored in the program. Users can select all or part of the traits to generate the trait file for the Field Book App. This is useful when breeders already have a BIMS program and want to use the Field Book App later or when multiple devices to collect data with Field Book are used.

**Figure 16.**
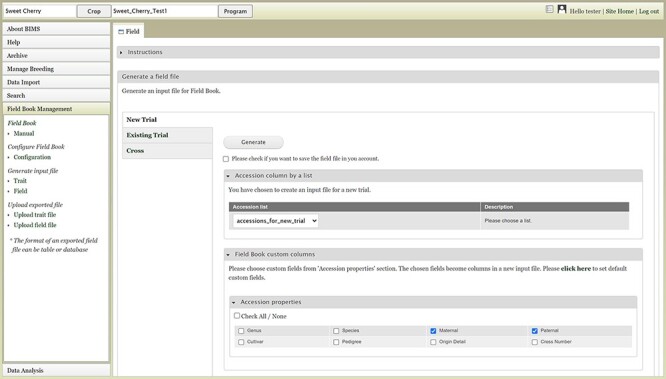
BIMS interface where users can create input files for the Field Book App.

The Field link under the *Generate input file* section opens a page with three choices: New Trial, Existing Trial and Cross (Figure 16). The New Trial section lets users choose one of the accession lists that are generated and saved in the Search section, choose additional columns and generate the field file. Users will still need to add a unique identifier, primary order and secondary order in the field file. The Existing Trial section can be used when users want to generate a newly updated field file for the existing trial. One breeding program often uses multiple devices for phenotyping, and this functionality can be used to merge the data. Breeders can periodically load the Field Book exported files to BIMS and then recreate an input file with an indication (e.g. a trait) distinguishing accessions already phenotyped. The Cross section allows breeders to automatically generate progeny names and generate an input file for a new cross. Breeders can upload a file with the cross name, row and the number of progeny for each row for BIMS to generate a Field Book input file with new progeny names. Compatibility between BIMS and Field Book app has exponentially increased the efficiency and efficacy of breeding programs as direct upload of data collected with Field Book app to BIMS enables instant access to the collected data.

### Data archive

Data in BIMS programs are backed up nightly on-site and off-site for security. The Archive section of the accordion menu, however, provides functionality for breeders to archive data at any time. In the Manage Archive page, users can click the Archive Data button to save the entire data in the program in the BIMS Excel file. The file is displayed in the Archives section on the left and when the file name is selected users can see the details and/or download in the Archive Details section on the right. All archived files can be accessed in the users' account page. When users want to download only certain types of data, they can expand Advanced options and choose the data types that they want to download.

### Customization

**Figure 17.**
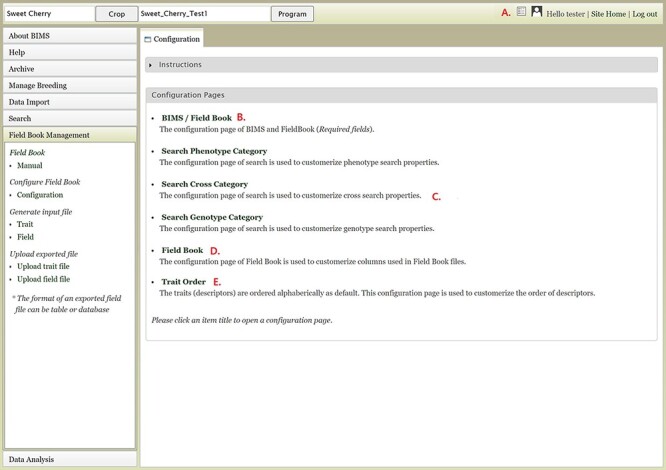
Configuration page of BIMS A. Hyperlink to the configuration page that is shown on top right of each BIMS page. B. BIMS/Field Book link In the configuration page. C. Links to tabs where users can modify the categories in search pages for phenotype, genotype and cross. D. Field Book link in the configuration page where users can choose traits, accession properties, or sample properties to be displayed in the Generate Field Book Input file section. E. Trait Order link in the configuration page.

Customization is one of the very important and unique features of BIMS. In addition to being able to add new traits at any time, BIMS offers various ways to customize the data management. As described previously, the customization can be done following the links in various pages under the accordion menu; users can access them through the configuration page link on the top right of each BIMS page (Figure 17A). The BIMS/Field Book link in the configuration page (Figure 17B) opens a tab where users can change the column names required for the Field Book App input file, such as accession, unique identifier, primary order and secondary order. Users can also add custom columns in templates to store their various metadata unique to their breeding program. Those custom columns can be selected to be used as search categories as well through the Search Phenotype/Cross/Genotype Category links (Figure 17C). The Field Book link on the configuration page (Figure 17D) lets users choose which traits, accession properties or sample properties will be displayed in the Generate Field Book Input file section and resulting Field Book input file. Finally, the Trait Order link in the configuration page lets users change the order of traits displayed in the Manage Breeding page (Figure 17E).

## Conclusions

We described BIMS, a free, secure online BMS that allows breeders to store, manage, archive and analyze their private breeding program data. BIMS allows management of both phenotype and genotype data and the various metadata for each breeding program. To our knowledge, BIMS is the only public domain breeding database system that allows crop breeders to combine publicly available breeding data within their own private breeding data. It is housed in the community database, but users are in complete charge of managing their data from creating a program, controlling access permissions, loading the data and editing the data. The connectivity to the community database provides the opportunity to import publicly available breeding data to their private program. Breeders can create and manage multiple breeding programs in BIMS so they can create one for a collaborative breeding project where the data can be shared among collaborators and another for their private data. Data templates are useful to incorporate historical breeding data into BIMS as well as the current breeding data. The presence of breeders’ data in the database will enable breeders to have increased control over internal data-sharing with collaborators and external release of their data to the public should they desire. Data-sharing and combining public breeding data with private breeding programs should increase the standardization of data. Currently, five community databases that support 26 tree fruit, tree nut, berry, pulse and fiber crops have implemented BIMS. It is expected that BIMS will support more crops with databases built using the Tripal platform, as the code is freely available and the BIMS team provides support for adoption. The adoption of BIMS with these functionalities by breeders of a wide variety of crops is likely to promote historic breeding data preservation, collaboration among breeders, data standardization and utilization of publicly available data and tools, in addition to increasing the effectiveness of each breeding program.

## Limitations and future development

Limitations of the current BIMS include the management of image data and lack of complex analysis functionalities. We are currently working on the management of image data, and we anticipate it to be completed by the end of this year. As part of NRSP10 project, we will add functionality for GWAS, to be completed by 2024. Current development also includes making BIMS compliant with the breeding API, BrAPI (https://www.brapi.org/). BrAPI is a web service API specification for exchanging plant phenotype and genotype data between crop breeding applications. This will allow users to send and receive data from other BrAPI compliant resources including the Field Book App and various analysis tools under development in the GOBii project (17). The work is already completed, and the updated BIMS has been implemented in one of our databases. We will make the updated code available after the functionality has been tested by collaborating breeders. We will also develop more search and download interfaces for genotype data and implement more breeding decision tools that utilizes both phenotype and genotype data. We are also planning to implement a BIMS instance on the BIMS website (www.breedwithbims.org) so that breeders without a community crop database can use BIMS.
